# Cysteinyl Leukotriene Receptor-1 Antagonists as Modulators of Innate Immune Cell Function

**DOI:** 10.1155/2014/608930

**Published:** 2014-05-25

**Authors:** A. J. Theron, H. C. Steel, G. R. Tintinger, C. M. Gravett, R. Anderson, C. Feldman

**Affiliations:** ^1^Medical Research Council Unit for Inflammation and Immunity, Department of Immunology, Faculty of Health Sciences, University of Pretoria, P.O. Box 2034, Pretoria 0001, South Africa; ^2^Tshwane Academic Division of the National Health Laboratory Service, Pretoria 0001, South Africa; ^3^Division of Pulmonology, Department of Internal Medicine, Charlotte Maxeke Johannesburg Academic Hospital and Faculty of Health Sciences, University of the Witwatersrand, Johannesburg 2193, South Africa

## Abstract

Cysteinyl leukotrienes (cysLTs) are produced predominantly by cells of the innate immune system, especially basophils, eosinophils, mast cells, and monocytes/macrophages. Notwithstanding potent bronchoconstrictor activity, cysLTs are also proinflammatory consequent to their autocrine and paracrine interactions with G-protein-coupled receptors expressed not only on the aforementioned cell types, but also on Th2 lymphocytes, as well as structural cells, and to a lesser extent neutrophils and CD8^+^ cells. Recognition of the involvement of cysLTs in the immunopathogenesis of various types of acute and chronic inflammatory disorders, especially bronchial asthma, prompted the development of selective cysLT receptor-1 (cysLTR1) antagonists, specifically montelukast, pranlukast, and zafirlukast. More recently these agents have also been reported to possess secondary anti-inflammatory activities, distinct from cysLTR1 antagonism, which appear to be particularly effective in targeting neutrophils and monocytes/macrophages. Underlying mechanisms include interference with cyclic nucleotide phosphodiesterases, 5′-lipoxygenase, and the proinflammatory transcription factor, nuclear factor kappa B. These and other secondary anti-inflammatory mechanisms of the commonly used cysLTR1 antagonists are the major focus of the current review, which also includes a comparison of the anti-inflammatory effects of montelukast, pranlukast, and zafirlukast on human neutrophils *in vitro*, as well as an overview of both the current clinical applications of these agents and potential future applications based on preclinical and early clinical studies.

## 1. Introduction

Cysteinyl leukotrienes (cysLTs), specifically LTs C_4_, D_4_, and E_4_, are generated predominantly by cells of the innate immune system following exposure to allergens, proinflammatory cytokines, and other types of receptor-dependent stimuli. The resultant mobilization of Ca^2+^ from intracellular and extracellular reservoirs leads to activation of cytosolic phospholipase A_2_ (cPLA_2_), as well as other types of PLA_2_ enzymes, which cleave arachidonic acid from membrane phospholipids, which is a prerequisite for generation of cysLTs [1–3]. Acting in concert with perinuclear membrane 5′-lipoxygenase- (5-LO-) activating protein (FLAP), arachidonate is oxygenated by 5-LO to LTA_4_, which undergoes sequential conversion to LTC_4_, LTD_4_, and LTE_4_ mediated by the enzymes LTC_4_ synthase, *γ*-glutamyl leukotrienase, and LTD_4_ dipeptidase, respectively [1–3].

These cysLTs are then available to interact with specific cysLT receptors (cysLTRs), namely, cysLTR1 and cysLTR2, expressed on the outer membrane of immune/inflammatory cells and structural cells. The former is the more widely distributed of the two types of receptor, being expressed on a range of cells of the innate immune system including basophils, mast cells, dendritic cells, eosinophils, and monocytes/macrophages, as well as B cells and CD4^+^ T cells, and to a lesser extent on neutrophils and CD8^+^ cells [1–3]. CysLTR1 is also expressed on various types of structural cell, including airway smooth muscle, epithelial, and endothelial cells, as well as fibroblasts [1–3]. LTD_4_ has the highest and LTE_4_ the lowest affinity for the cysLTR1. CysLTR2, as well as other selective and more promiscuous types of cysLTR, have a more limited cellular distribution, and although discrete functions of these are emerging, these will not be addressed in the current review which is focused on cysLTR1 antagonists.

Interaction of cysLTs with cysLTR1 expressed on immune/inflammatory cells, airway smooth muscle, and other types of structural cell is intimately involved in many aspects of the immunopathogenesis of bronchial asthma, including chronic eosinophilic airway inflammation, bronchoconstriction, bronchial hyperresponsiveness, mucus hypersecretion, and airway remodeling [1]. Recognition of the cysLT/cysLTR1 axis in the immunopathogenesis of bronchial asthma, as well as allergic rhinitis, provided the impetus for development of selective antagonists of cysLTR1. Pranlukast was introduced for clinical application in Japan in 1995 and is currently marketed in this and several other Asian countries. Two others, montelukast and zafirlukast, were subsequently developed, receiving FDA approval in 1998 and 1999, respectively. Montelukast, probably due to its once daily dosing schedule and safety and efficacy profiles is the most widely prescribed cysLTR1 antagonist in USA and Europe. These agents have found niche applications in the treatment of allergic rhinitis, exercise- and aspirin-induced asthma, and as add-on therapy in patients with asthma poorly controlled by inhaled corticosteroid (ICS) monotherapy or ICS in combination with long-acting *β*2-agonists [4, 5].

Topics to be covered in the following sections of this review include: (i) the cysLTR-dependent proinflammatory interactions of cysLTs with cells of the innate immune system, particularly neutrophils; (ii) cysLTR1-independent anti-inflammatory actions of the cysLTR1 antagonists which target neutrophils and monocytes/macrophages in particular; (iii) a comparison of the neutrophil-targeted, cysLTR1-independent, suppressive effects of montelukast, pranlukast, and zafirlukast; and (iv) current and potential applications of cysLTR1 antagonists.

## 2. CysLTR1-Dependent Proinflammatory Interactions of CysLTs with Cells of the Innate Immune System

The interaction of cysLTs with their type 1 counter-receptors on cells of the innate immune system results in the release of a series of inflammatory mediators which, in addition to exacerbating bronchial hyperresponsiveness, mucus hypersecretion, and airway remodeling, also drive Th2-cell-mediated eosinophilic airway inflammation. Cells of the innate immune system which undergo activation on exposure to cysLTs include mast cells/basophils, monocytes/macrophages, and myeloid dendritic cells, all of which also produce cysLTs. Neutrophils, on the other hand, respond only modestly to cysLTs.

This is surprising since neutrophils, as described in later sections of this review, are extremely sensitive to the suppressive effects of cysLTR1 antagonists. The proinflammatory interactions of cysLTs with mast cells [6–8], monocytes/macrophages [9–16], eosinophils [17–20], dendritic cells [14, 21, 22], and neutrophils [23–25], all of which are antagonized by montelukast and pranlukast or zafirlukast, are summarized in Table 1. Proinflammatory interactions of cysLTs with neutrophils are covered in greater detail below.

### 2.1. Neutrophils

Although neutrophils do not produce cysLTs they do, however, express cysLTR1, albeit at lower levels than the aforementioned cell types [2]. Exposure of human neutrophils to LTC_4_ and LTD_4_ has been reported to result in modest activation of Ca^2+^ mobilization and production of nitric oxide in comparison with LTB_4_ and other potent neutrophil chemoattractants [23, 24]. On the other hand adhesion to vascular endothelium, release of granule proteases, and NADPH oxidase-mediated generation of superoxide anion are all unaffected following exposure of neutrophils to LTC_4_ and LTD_4_ [23–25]. Of greater potential significance, however, is the “priming” interaction of cysLTs with human neutrophils, which sensitizes these cells for increased production of ROS and release of MMP-8 following exposure of the cells to the chemoattractant, N-formyl-L-methionyl-L-leucyl-L-phenylalanine (fMLP) [25]. Both the direct activating and sensitizing interactions of cysLTs with human neutrophils are attenuated by either an experimental cysLTR1 antagonist (SK*α*F 104353) [23, 24] or montelukast [25].

## 3. CysLTR1-Independent Anti-Inflammatory Mechanisms of CysLTR1 Receptor Antagonists

In addition to anti-inflammatory activity achieved via blockade of cysLTR1, montelukast, pranlukast, and zafirlukast have also been reported to possess anti-inflammatory properties, primarily targeting neutrophils and monocytes/macrophages, which are independent of cysLTR1 antagonism. In this setting, the anti-inflammatory effects of these agents are achieved at concentrations somewhat higher than those required to achieve maximal cysLTR1 blockade, but which are nonetheless close to the peak serum concentrations attained during chemotherapy with these agents. In the case of montelukast, this agent at a concentration of 0.1 *μ*M effectively suppresses Ca^2+^ mobilization following exposure of neutrophils to LTD_4_ [25], while concentrations of ≥0.25 *μ*M are required to exert the cysLTR1-independent effects described below [26]. Peak serum concentrations of 0.5–1 *μ*M are attained following oral administration of montelukast in the therapeutic setting [27, 28].

Several experimental strategies have been used to ensure veracity of interpretation of the cysLTR1-independent anti-inflammatory activities of the cysLTR1 antagonists described below. These include (i) addition of inhibitors of the LT-generating enzyme, 5-lipoxygenase (5-LO), in the various assay systems to eliminate the potentially complicating effects of generation of cysLTs by target cells and/or contaminating cells in the cell suspensions and (ii) inactivation of expression of cysLTR1 on target cells using gene knockout strategies.

Several mechanisms underpinning the cysLTR1-independent anti-inflammatory activities of cysLTR1 antagonists have been described in detail elsewhere [29] and are updated in the current review together with inclusion of several more recently described mechanisms. These are (i) inhibition of 5-LO, resulting in attenuation of production not only of cysLTs but also of LTB_4_ [26, 30, 31]; (ii) nonspecific inhibition of cyclic nucleotide phosphodiesterases (PDEs) [26, 32], resulting in increased levels of 3′,5′-cyclic adenosine monophosphate (cAMP), a major regulator of the proinflammatory activities of cells of the innate immune system [33]; (iii) inhibition of the activity of the transcription factor, NF*κ*B [29, 34–36]; (iv) inhibition of prostaglandin E synthase [37]; (v) inhibition of eosinophil adhesion and migration [38, 39]; and (vi) antagonism of purinergic P2Y receptors [31].

### 3.1. ****5-Lipoxygenase

The inhibitory effects of montelukast and zafirlukast on the production of LTs by both neutrophils and monocytes/macrophages are well documented [26, 30, 31] and are achieved at concentrations close to the peak serum concentrations of montelukast. Notwithstanding attenuation of production of cysLTs, inhibition of generation of the potent neutrophil chemoattractant, LTB_4_, by these agents represents a potential strategy to control hyperreactivity of the corticosteroid-resistant neutrophil. Although montelukast was found to have direct inhibitory effects on 5-LO, this was only evident at very high concentrations of this agent [30], well above peak serum levels, suggesting the existence of an alternative mechanism of inhibition as described below.

### 3.2. Cyclic AMP Phosphodiesterases

In addition to inhibition of 5-LO, montelukast at concentrations of ≥0.5 *μ*M has also been reported to inhibit the production of ROS and release of elastase by chemoattractant-activated neutrophils, as well as expression of the *β*2-integrin, CR3 [26, 40]. Inhibition of these neutrophil Ca^2+^-dependent activities was associated with increased clearance of cytosolic Ca^2+^ in the setting of increased concentrations of intracellular cAMP [26, 40]. Accelerated clearance of cytosolic Ca^2+^ and downregulation of neutrophil proinflammatory activity were attributed to activation of cAMP-dependent protein kinase (PKA), which, in turn, promotes restoration of Ca^2+^ homeostasis by several mechanisms, including increased efficiency of Ca^2+^ sequestration/resequestration into stores [29]. In addition, PKA has also been reported to inhibit the activation of 5-LO [41].

The potentiating effects of montelukast on cAMP, restoration of Ca^2+^ homeostasis, and attenuation of neutrophil proinflammatory activity were found to be closely correlated with direct, nonspecific, inhibitory effects of montelukast on cyclic nucleotide PDEs [26]. These effects of montelukast on cyclic nucleotide PDEs have recently been confirmed by others using a model of salbutamol-mediated desensitization of *β*2-adrenoreceptors in airway smooth muscle cells, as well as in an isolated PDE preparation [32].

### 3.3. NF Kappa B

Pranlukast and montelukast have been reported to inhibit the activation of the transcription factor, NF*κ*B, in a variety of cell types including monocytes/macrophages, T cells, epithelial cells, and endothelial cells (reviewed in [29, 34]). The consequence is interference with the generation of various proinflammatory proteins, including IL-8. However, the mechanism underpinning the inhibitory effects of the cysLTR1 antagonists on the activity of the transcription factor remain uncertain. While interference with nuclear translocation has been described [34], others have reported that the inhibitory effects of montelukast on NF*κ*B activity in monocytes/macrophages are not achieved via inhibition of DNA binding [35]. In this latter setting, treatment of the cells with montelukast was associated with significant inhibition of histone acetyltransferase (HAT) activity, consistent with impaired activation of the transcriptional coactivator proteins which facilitate histone acetylation and unwinding of chromatin, events which precede and are a prerequisite for gene transcription [35]. Interestingly, PKA has also been reported to negatively regulate NF*κ*B-activated gene transcription in monocytes/macrophages and endothelial cells without affecting interaction of the transcription factor with DNA [36], consistent with the involvement of cAMP in montelukast-mediated interference with NF*κ*B.

### 3.4. Prostaglandin Synthase

Montelukast, pranlukast, and zafirlukast, at IC50 concentrations between 2 and 4 *μ*M, have been reported to inhibit the synthesis of prostaglandin (PG) E_2_ by isolated, lipopolysaccharide-activated human leukocytes, as well as by various cytokine-exposed cancer cell lines* in vitro*, apparently by direct inhibition of microsomal PGE synthase-1 [37]. Aside from cysLTR1-independent anti-inflammatory effects, these observations may also be of significance in relation to the antitumor activities of cysLTR1 antagonists [42]. They should, however, be viewed in the context of the study by Woszczek et al. who failed to detect inhibitory effects of either montelukast or zafirlukast on the production of PGE_2_ by stimulated human monocytes/macrophages* in vitro* [31].

### 3.5. Eosinophil Activity

As mentioned in a previous review [29], montelukast, at therapeutically relevant concentrations and above, has been reported to interfere with (i) the *α*_4_ *β*_1_ (*β*_1_-integrin)-mediated binding of eosinophils to VCAM-1 [38] and (ii) migration activated by the chemoattractant, 5-oxo-6,8,11,14-eicosatetraenoic acid, which was associated with impaired expression of the urokinase plasminogen receptor and release of MMP-9 [39].

### 3.6. P2Y Receptors

This aspect of the cysLTR1-independent anti-inflammatory activity of cysLTR1 antagonists has recently been reviewed elsewhere [29]. Suffice it to say that montelukast, pranlukast, and zafirlukast, at micromolar concentrations, have been reported to antagonize the interactions of nucleotides with their P2Y counter-receptors on human monocytes/macrophages, resulting in attenuation of synthesis of IL-8 [29, 31]. However, given the relatively high micromolar concentrations of the cysLTR1 antagonists at which these effects are achieved, their therapeutic significance remains uncertain [31].

### 3.7. Adenosine Monophosphate- (AMP-) Activated Protein Kinase

More recently pranlukast, at supratherapeutic concentrations of up to 50 *μ*M, was found to increase the activity of AMP-activated protein kinase in a canine kidney cell line (MDCK cells) by a putative cysLTR1-independent mechanism involving activation of calcium/calmodulin-dependent protein kinase kinase beta [43]. The effects of montelukast and zafirlukast were complicated by cytotoxicity at the high concentrations used [43]. Notwithstanding the high concentration of pranlukast required to achieve these effects, this mechanism, if operative in cells of the innate immune system, represents an additional mechanism of cysLTR-independent anti-inflammatory activity. This is because AMP-activated protein kinase suppresses the activity of NF*κ*B in human umbilical vein endothelial cells [44].

**Figure 1:**
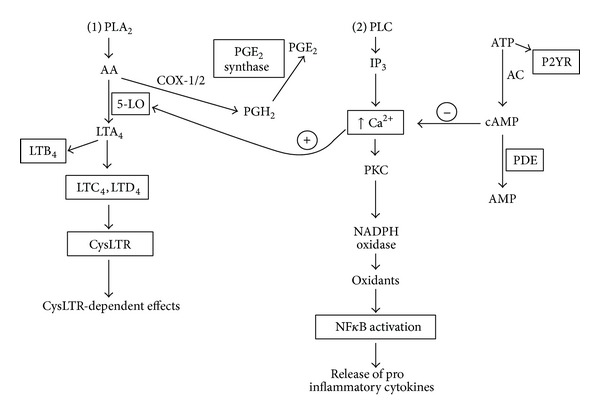
Cysteinyl leukotriene receptor-1-independent and -dependent anti-inflammatory activities of montelukast, pranlukast, and zafirlukast. The intracellular signalling pathways are depicted with the targets of the cysLTR1 antagonists indicated by means of a rectangle. Phospholipase A_2_ (PLA_2_) activation that is represented in pathway 1 during which membrane phospholipids are converted to arachidonic acid (AA), which is subsequently metabolized to either leukotrienes (LTA_4_, LTB_4_, and LTC_4_) or prostaglandins (PGH_2_) by 5-lipoxygenase (5-LO) and COX-1/2 enzymes, respectively. PGH_2_ is converted to PGE_2_ by PGE_2_ synthase. Phospholipase (PLC) activation is represented in pathway 2 with the generation of inositol triphosphate (IP_3_) which releases cytosolic Ca^2+^ from storage vesicles with subsequent downstream activation of PKC, NADPH oxidase, 5-LO, and NF*κ*B, with release of proinflammatory cytokines. ATP binds to purinergic receptors (P2YR) and is also converted to cyclic AMP by adenylate cyclase (AC) which is degraded by intracellular phosphodiesterases (PDE). Cyclic AMP downregulates the Ca^2+^-mediated activation of 5-LO and NADPH oxidase. Inhibition of cysLTR1 receptors attenuates the receptor-dependent effects of cysteinyl leukotrienes.

These cysLTR1-independent anti-inflammatory activities of montelukast, pranlukast, and zafirlukast are summarized in Table 2 and Figure 1.

## 4. Comparative Anti-Inflammatory Effects of Montelukast, Pranlukast, and Zafirlukast on Human Neutrophils* In Vitro*

Although the cAMP-mediated suppressive effects of montelukast have been documented previously [26, 29, 40], relatively little is known about the anti-inflammatory interactions of pranlukast and zafirlukast with these cells. This issue has been addressed in the current section of this review, specifically a comparison of the effects of the 3 cysLTR1 antagonists on the production of ROS and LTB_4_ by chemoattractant-activated human neutrophils* in vitro*, as well as the release of elastase from these cells in the context of nonspecific PDE inhibitory activity.

Neutrophils with their arsenal of indiscriminate reactive oxygen species (ROS) and proteases pose a potential threat to bystander host cells and tissues in the vicinity of inflammatory reactions. Few currently available therapeutic agents, including corticosteroids, effectively control the harmful proinflammatory activities of neutrophils [45, 46]. We have previously found that montelukast, primarily a cysteinyl leukotriene (CysLT_1_) receptor antagonist, exhibited secondary, neutrophil-directed anti-inflammatory properties, which appeared to be cAMP-mediated [26].

We have recently compared the effects of montelukast, pranlukast, and zafirlukast at therapeutically relevant concentrations on several Ca^2+^-dependent, proinflammatory activities of isolated human neutrophils. Montelukast was provided by Merck Research Laboratories (Rahway, NJ, USA), zafirlukast by AstraZeneca (Johannesburg, South Africa), and pranlukast was purchased from the Cayman Chemical Company (Ann Arbor, MI, USA). All 3 agents were dissolved to a stock concentration of 10 mM in dimethylsulfoxide (DMSO) and used at final concentrations of 0.25–2 *μ*M. The final concentration of DMSO in each assay was 0.1% and appropriate solvent controls were included with each experimental system. Unless indicated, all other chemicals and reagents were purchased from Sigma-Aldrich (St Louis, MO, USA).

The study was approved by the Faculty of Health Sciences Research Ethics Committee of the University of Pretoria, Pretoria, South Africa, and prior informed consent was obtained from all blood donors. Neutrophils were isolated from heparinized venous blood (5 units of preservative-free heparin per mL of blood) from healthy adult volunteers as described previously [40] and resuspended to 1 × 10^7^ cells per mL in phosphate-buffered saline (0.15 M, pH 7.4) and held on ice until used. For the assays described below, the cells were suspended in Hanks' balanced salt solution (indicator-free; pH 7.4; Highveld Biological, Johannesburg, South Africa).

The results of each series of experiments are presented as the mean values ± the standard errors of the means (SEMs), where *n* equals the number of different donors. Levels of statistical significance were determined by comparing the absolute values for each drug-treated system with the corresponding values for the relevant drug-free control systems for each assay using the Wilcoxon matched pairs test.

### 4.1. Superoxide Production

**Figure 2:**
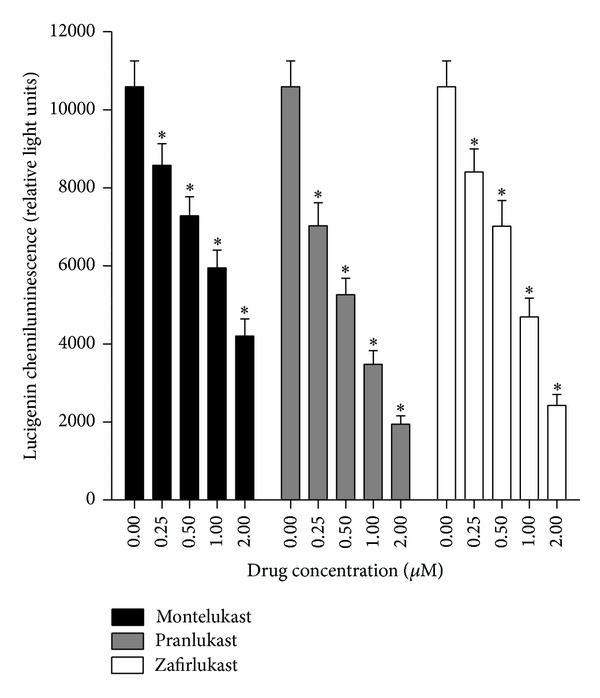
Effects of montelukast, pranlukast, and zafirlukast (0.25–2 *μ*M) on the lucigenin-enhanced chemiluminescence responses of neutrophils activated by N-formyl-L-methionyl-L-leucyl-L-phenylalanine (fMLP, 1 *μ*M). The results are expressed as the mean peak chemiluminescence values in relative light units measured at 30–50 s after addition of fMLP and vertical lines show SEM (*n* = 6 with 2 replicates for each drug concentration and control system in each experiment). The absolute values for unstimulated neutrophils and for cells activated with fMLP in the absence of the drugs were 2099 ± 223 and 10594 ± 660 relative light units, respectively. *P < 0.05 for comparison with the fMLP-activated, drug-free control system.

This was measured using a lucigenin- (bis-N-methylacridinium nitrate-) enhanced chemiluminescence (LECL) procedure as previously described [26]. Following pretreatment of the neutrophils with the 3 agents, the cells were activated with the chemoattractant, N-formyl-methionyl-leucyl-phenylalanine (fMLP, 1 *μ*M), and LECL responses recorded as described [26]. The results, which are shown in Figure 2, indicate that all 3 agents at concentrations of 0.25–2 *μ*M caused essentially comparable, dose-related inhibition of the generation of superoxide production which achieved statistical significance at all the concentrations tested.

### 4.2. Elastase Release

**Figure 3:**
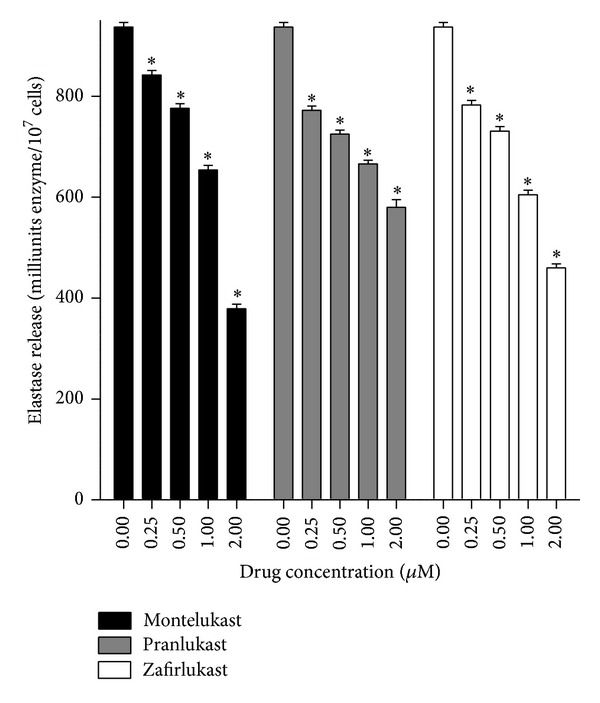
Effects of montelukast, pranlukast, and zafirlukast (0.25–2 *μ*M) on the release of elastase from neutrophils activated with N-formyl-L-methionyl-L-leucyl-L-phenylalanine (1 *μ*M)/cytochalasin B (1 *μ*M) (fMLP/CB). The results (*n* = 6 with 5 replicates for each drug concentration and control system in each experiment) are expressed as the mean values for total extracellular elastase (milliunits/10^7^ cells) and vertical lines show SEM. The absolute values for the unstimulated control system and for cells activated with fMLP/CB were 33 ± 1 and 937 ± 9 milliunits elastase/10^7^ cells. *P < 0.05 for comparison with the drug-free control system.

Neutrophil degranulation was measured according to the extent of release of the primary granule enzyme, elastase, as previously described [40]. Supernatants of cells, pretreated with the 3 test agents and activated with fMLP/cytochalasin B (F/CB, 1 *μ*M/1 *μ*M), were assayed for elastase using a standard colorimetric method [40]. The results of these are shown in Figure 3. Montelukast, pranlukast, and zafirlukast at concentrations of 0.25–2 *μ*M caused essentially comparable, dose-related inhibition of the generation of elastase release which achieved statistical significance at all the concentrations tested.

### 4.3. Leukotriene B_4_ (LTB_4_)

**Figure 4:**
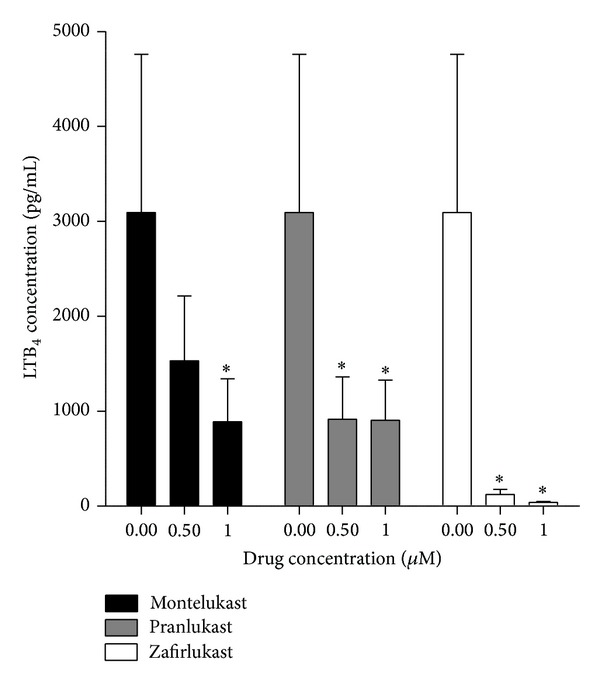
Effects of montelukast, pranlukast, and zafirlukast (0.5 and 1 *μ*M) on the production of LTB_4_ by PAF-activated neutrophils. The results are presented as the mean values for total extracellular LTB_4_ (pg/mL) and vertical lines show SEM (*n* = 9 with 2 replicates for each drug concentration and control system in each experiment). The absolute values for the unstimulated control system and for cells activated were 21 ± 5 and 3092 ± 1669 pg LTB_4_/mL, respectively. *P < 0.05 for comparison with the drug-free control system.

A competitive binding immunoassay procedure (Correlate-EIA; Assay Designs Inc., Ann Arbor, MI, USA) was used to measure LTB_4_ in the supernatants of neutrophils activated with the chemoattractant, platelet-activating factor (PAF, 200 nM), in the absence and presence of the leukotriene receptor antagonists (0.5 and 1 *μ*M). These results are shown in Figure 4. All 3 test agents caused statistically significant, dose-related inhibition of LTB_4_ production by PAF-activated neutrophils with zafirlukast being most potent.

### 4.4. PDE Activity

**Figure 5:**
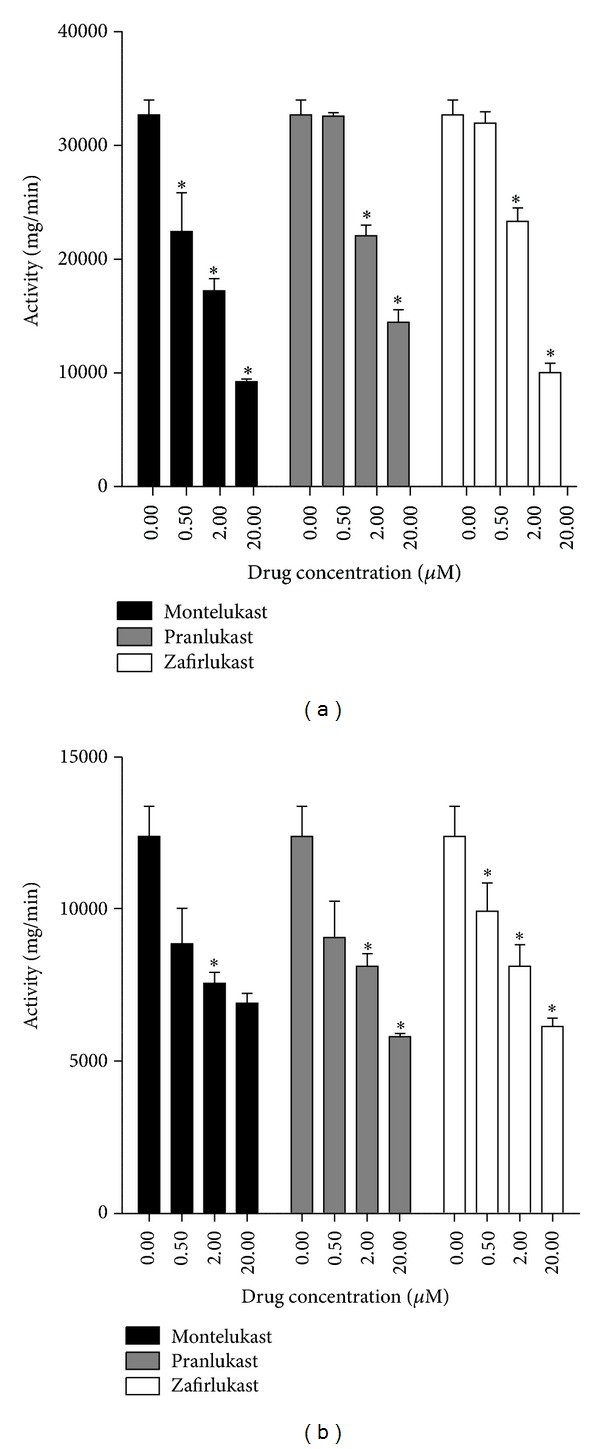
Effects of montelukast, pranlukast, and zafirlukast (0.5–20 *μ*M) on cAMP- (a) and cGMP- (b) phosphodiesterase (PDE) activities in neutrophil cytosol. The results are presented as the mean enzyme activities, and vertical lines show SEM (*n* = 2–5, with three to four replicates for each drug concentration and control system in each experiment). *P < 0.05 for comparison with the corresponding drug-free control system.

The PDE inhibitory activity of montelukast, pranlukast, and zafirlukast was assessed using a scintillation proximity assay (SPA, Amersham Biosciences, UK) as described previously [26]. Reaction mixtures contained neutrophil cytosol, as a source of PDE, [^3^H]cAMP or [^3^H]cGMP, in the absence and presence of the cysLTR1 antagonists (0.5–20 *μ*M) [26]. As shown in Figure 5, the cysLTR1 antagonists at concentrations of 0.5–20 *μ*M caused dose-related inhibition of both cAMP- and cGMP-PDE activity which in all cases achieved statistical significance at concentrations of ≥2 *μ*M. These findings demonstrate that all 3 cysLTR1 antagonists possess nonspecific PDE inhibitory activity.

### 4.5. Cellular ATP Levels

To determine the effects of pranlukast and zafirlukast (2 *μ*M) on neutrophil viability, intracellular ATP concentrations were measured in cell lysates (1 × 10^6^ cells/mL) following exposure of the cells to the drugs for 10 min at 37°C, using a luciferin/luciferase chemiluminescence procedure [47]. As reported previously for montelukast [26], treatment of neutrophils with these agents did not affect neutrophil ATP levels; the values for the control and pranlukast- and zafirlukast-treated cells were 6.4 ± 0.34, 6.84 ± 0.50, and 6.72 ± 0.49 pmols ATP/10^7^ cells, respectively (*n* = 2, with seven replicates for each system in each experiment).

### 4.6. Comment

The results of the aforementioned experiments demonstrate that montelukast, pranlukast, and zafirlukast, at concentrations within the therapeutic range and above, caused significant, dose-related inhibition of superoxide generation, as well as production of LTB_4_ and release of elastase, by activated neutrophils. The effects of the 3 agents were mostly comparable although zafirlukast was more potent than the other agents with regard to its inhibitory effect on LTB_4_ production. The observed effects were not due to cytotoxicity as the drugs did not affect levels of cellular ATP.

Although not shown, the inhibitory effects of all 3 test agents on the generation of ROS by chemoattractant-activated neutrophils were unaffected by the inclusion of the 5-LO inhibitor, MK886 (0.5 *μ*M), in the assay system, consistent with lack of involvement of cysLTR1 antagonism. From a mechanistic perspective, all 3 cysLTR1 antagonists, at concentrations virtually superimposable on those which suppressed the production of inflammatory mediators by neutrophils, were found to possess nonspecific PDE inhibitory activity. Although the existence of other mechanisms of cysLTR1-independent anti-inflammatory activity cannot be excluded, nonspecific PDE inhibitory activity is likely to underpin the anti-inflammatory effects of the cysLTR1 antagonists. In this setting, elevations in intracellular cAMP promote downregulation of neutrophil proinflammatory activity via accelerated clearance of Ca^2+^ from the cytosol of activated cells [26, 40]. In support of this proposed mechanism, we have also observed that all 3 cysLTR1 antagonists, at concentrations which inhibit PDE activity and production/release of inflammatory mediators, also promote accelerated clearance of Ca^2+^ from the cytosol of activated neutrophils (not shown).

Although the 3 test agents have been found to effectively suppress the proinflammatory activities of neutrophils* in vitro* by a cysLTR1-independent mechanism, the clinical significance, as well as the aspects of molecular structure which confer nonspecific PDE inhibitory activity on these agents, remains to be established.

The final section of this review is focused on the current clinical applications of cysLTR1 antagonists, as well as potential future clinical indications.

## 5. Clinical Applications of CysLTR1 Antagonists

CysLTR1 antagonists have a significant role to play in airway disorders, in particular allergic rhinitis (AR) and/or asthma [48, 49]. Studies have also suggested that they may have potential benefit in other disorders that are often associated with asthma, as well as in a number of conditions that are not linked to asthma [49]. This section will review the role of cysLTR1 antagonists in allergic rhinitis (AR) and asthma, including pediatric, adult, and elderly patients, as well as the various subsets of asthmatic patients, such as those with AR, those with aspirin-sensitive asthma and those with exercise-induced asthma [50]. Most well studied has been the role of montelukast in the management of these various conditions, which will therefore be the focus of this section. This is followed by a brief consideration of potential, albeit unproven, clinical applications of cysLTR1 antagonists.

### 5.1. Allergic Rhinitis

As mentioned earlier, cysLTR1 antagonists, particularly montelukast, have been registered in a number of countries for the treatment of AR and are considered to be a suitable alternative to other available therapies. The conclusions of a number of studies, as well as an evidence based review, is that montelukast is superior to placebo in alleviating symptoms of both seasonal and perennial AR, that as monotherapy it is equivalent to the anti-histamine, loratadine (second generation H1 receptor antagonist), but is not as efficacious as intranasal fluticasone propionate and that when combined with loratadine or cetirizine has superior efficacy to each of these agents alone, producing results similar to those of intranasal corticosteroids [50–52]. In one study comparing an intranasal corticosteroid, a cysLTR1 antagonist and a topical antihistamine, the authors concluded that montelukast, because of its systemic effects, is more efficacious at relieving symptoms beyond the nasal cavity [53]. In one study of montelukast, given either alone or combined with desloratadine or levocetirizine in patients with persistent AR, a significant improvement of nasal symptoms was documented in the first 24 hours, which gradually increased up to 6 weeks [54]. Another study of patients with seasonal AR documented montelukast 5 mg or 10 mg once daily to be as safe and effective as pranlukast (450 mg/day) [55].

### 5.2. Asthma

#### 5.2.1. Chronic Asthma

The cysLTs have, at least in some patients, been shown to play a leading role in the various pathological airway manifestations of asthma that lead to symptoms, including the occurrence of bronchoconstriction, formation of edema, and hypersecretion of mucus [56]. It is therefore not surprising that the cysLTR1 antagonists have enjoyed a well-established role in the treatment of patients with asthma for a considerable number of years, with efficacy and safety confirmed in a myriad of studies [3, 57–63]. The clinical benefits have been documented in various studies and reviews for montelukast [4, 64–66], zafirlukast [67–71], and pranlukast [72–78].

One area of contention is the exact role of cysLTR1 antagonists in the treatment algorithm for asthma, whether this should be as a monotherapy alternative to inhaled corticosteroids (ICS) or as an add-on therapy to corticosteroids instead of long-acting beta-agonists (LABA) and the relative efficacy of the cysLTR1 antagonists compared to these alternative treatment options [50, 79]. One recent “real-world” study in asthmatic patients, involving two parallel multicenter pragmatic trials, suggested that cysLTR1 antagonists may be equivalent to ICS as first-line monotherapy and equivalent to LABA as add-on therapy in patients with asthma not controlled on ICS alone, although the authors did recommend caution in the interpretation of the results because of the nature of the studies [80]. Interestingly, adherence to cysLTR1 antagonist therapy was better than the other agents used in those studies. Nevertheless, extensive review of the various studies has suggested, firstly, that ICS appear to have superior efficacy to antileukotrienes in many adults and children with asthma, and particularly those with moderate airway obstruction, such that guideline recommendations of ICS as preferred monotherapy appears appropriate [81, 82]. Secondly, review of data for add-on therapy for patients with asthma not adequately controlled on ICS alone suggests that while cysLTR1 antagonists do appear to have benefit, the efficacy of the addition of LABA may be greater [83–85]. However, it does appear that cysLTR1 antagonists may have a better long-term safety than LABAs [85]. Some experts have suggested that the choice of add-on therapy (either LABAs or cysLTR1 antagonists) should also be tailored to individual asthma patients (see also asthma phenotypes described below) [86].

#### 5.2.2. Asthma in Pediatrics

All of the licensed cysLTR1 antagonists are available in pediatric doses, but montelukast (registered in many areas from even 6 months) has been the most investigated. A myriad of studies and reviews attest to the potential benefits and safety of montelukast in pediatric asthma, although it is recognized that there are individual variations in response among the patients [87–96]. There is some debate as to the exact place of montelukast and other cysLTR1 antagonists in the management of pediatric patients, but it has been proposed that they may be used as an alternative to ICS as monotherapy in intermittent or mild persistent asthma, particularly in those who cannot or will not use ICS, or as add-on therapy for patients not controlled on ICS alone or as add-on therapy to reduce ICS doses in moderate or severe asthma, and in specific patient phenotypes (such as children aged 2–5 years to prevent frequent exacerbations and in those with concomitant AR) or in exercise-induced bronchospasm, or viral-induced wheeze [90, 92, 93, 95]. Although some studies have suggested that montelukast is an effective monotherapy controller for mild asthma in children, a recent systematic review comparing montelukast with ICS reported that the majority of studies indicated that ICS are as effective as or more effective than montelukast, and the authors concluded that ICS should remain the first-line treatment option for children with mild-moderate asthma [91, 96].

#### 5.2.3. Asthma in the Elderly

There have been relatively few studies of cysLTR1 antagonists in the elderly with asthma. However, what studies there are have suggested that these agents, particularly montelukast, are effective even in the elderly with severe asthma and appear overall to have equivalent benefit as add-on therapy to regular maintenance treatment as compared to younger patients [97, 98]. One problem with asthma treatment in the elderly is low adherence to therapy, and the easier route of administration of montelukast (oral agent versus inhaler) could be associated with improvement in overall treatment.

#### 5.2.4. Asthma and Allergic Rhinitis

While many patients with asthma have allergic rhinitis, it is also recognized that patients with AR are more likely in the future to develop asthma, so that the two conditions frequently coexist. It has been increasingly recognized that these two conditions appear, therefore, to be linked by being interacting manifestations of a common pathological mechanism; this association is often being described as “one airway, one disease” or “one linked airway disease” [52, 99–101]. Both montelukast and zafirlukast have been shown to be effective for the treatment of these two conditions when they occur either alone, as described above, or concomitantly [48, 52, 99–102].

#### 5.2.5. Acute Asthma

There has been interest in the use of both oral and intravenous cysLTR1 antagonists in the management of patients with acute asthma as an adjunct to standard care. In the case of intravenous montelukast, randomized studies in adults have shown greater efficacy than placebo, with a significant increase in forced expiratory volume in one second (FEV_1_) [103–105]; however, a similar study in children was unable to show benefit on FEV_1_, asthma symptoms, or the hospital course [106]. Similar randomized, double blind, placebo-controlled studies of oral montelukast in adult patients with acute asthma exacerbations, treated with standard of care, have documented only a higher peak expiratory flow (PEF) the morning after admission [107] or no benefit [108]. In children (5–15 years of age) the addition of a single dose of oral montelukast to standard treatment in acute moderate to severe asthma showed no additional clinical benefit [109]. Other investigators, using data from additional studies have questioned whether there may be a role for oral montelukast in asthma exacerbations [110, 111]. Nevertheless, a systematic review of the available data concluded that there was no evidence to support the routine use of oral cysLTR1 antagonists in acute asthma in either adults or children [112].

#### 5.2.6. Asthmatics Who Smoke

A study was undertaken to test the hypothesis that administration of montelukast (10 mg/day) would increase asthma control over 6 months, compared with placebo, in asthmatics that smoked [113]. The reason for performing such a study was due to the contention that smokers with asthma have a reduced response to corticosteroids. This was a parallel group study in which one additional arm was treated with fluticasone propionate (FP) (250 *μ*g). The main findings of the study were that montelukast therapy increased the mean percentage of asthma control days, as did FP, and while FP tended to have more benefit in patients with a pack-year smoking history <11 years, there was a tendency to greater benefit with montelukast in those with a smoking history >11 pack-years. Others have also suggested a greater benefit of montelukast in smokers, but clearly more data is required [114].

#### 5.2.7. Exercise-Induced Asthma (EIA)

Exercise-induced bronchospasm occurs commonly in asthmatic patients, both adult and children, sometimes as the only trigger of asthma and also occasionally in nonasthmatic patients. A number of pharmacological and nonpharmacological treatment approaches are recommended for the management of patients with EIA, which also include the cysLTR1 antagonists, especially montelukast or zafirlukast [5, 115–124]. Both montelukast and zafirlukast have been used either long term, particularly in asthmatic patients not controlled on baseline anti-asthma therapy, or even short term/acutely (e.g., single dose prior to exercise) and to provide useful protection against EIA, although there is some debate as to whether these agents are equivalent to or more or less effective than LABAs [5, 115–124].

#### 5.2.8. Aspirin-Intolerant Asthma

Intolerance to aspirin and other nonsteroidal anti-inflammatory agents (NSAIDs) may sometimes be a considerable clinical problem in patients with asthma, and following their discovery it became evident that the leukotrienes appeared to play an important role in these patients [125]. The mechanism appears to be related to cyclooxygenase inhibition by aspirin/NSAIDs, possibly resulting in excessive production of cysLTs [125]. Studies using cysLTR1 antagonists and other leukotriene modifiers have suggested that these agents may be of benefit in patients with aspirin-intolerant asthma [125, 126]. One randomized, double blind, placebo controlled study in aspirin-intolerant asthmatics, of whom 90% were already on moderate-to-high doses of corticosteroids, demonstrated a considerable improvement in asthma in the montelukast treated arm, which was unrelated to baseline LTE_4_ levels [126].

### 5.3. Other Disorders

It is also being increasingly recognized that cysLTR1 antagonists may have benefit in diseases beyond allergic rhinitis and asthma, some of which are described later, although none of these agents is currently registered for use in these conditions. One disease entity that has been particularly well studied is chronic obstructive pulmonary disease (COPD).

#### 5.3.1. Chronic Obstructive Pulmonary Disease (COPD)

There has been interest in whether leukotriene modifiers may have a role in the management of COPD, although data have been relatively limited [127]. However, studies with montelukast in both the short term [128] and long term [129] have suggested benefit in patients with COPD. In the former study, which was a randomized, prospective, single-blind, controlled study in 117 patients with COPD, randomized to ipratropium, formoterol and montelukast, or ipratropium and formoterol alone, showed benefits of montelukast, in addition to standard therapy, improved lung function testing, dyspnoea score, and quality of life [128]. The long-term study in COPD patients showed benefits of addition of montelukast to standard therapy including a significant improvement in symptoms, reduction in corticosteroid and bronchodilator therapy, and reduction in emergency room visits and hospitalizations or duration of hospitalizations for exacerbations, although there was no effect on lung function [129]. A more recent study in elderly patients with COPD in whom montelukast 10 mg/day was added documented a decrease in serum levels of LTB_4_ and interleukin-8 (IL-8) as well as a decrease in the number of outpatient visits and the number and duration of hospitalizations [130]. Montelukast was also documented to attenuate hypertonic saline-induced airway responses in patients with COPD [131]. In a number of studies, zafirlukast has been shown to have beneficial effects on lung function in patients with COPD, including those with severe airflow limitation, even in the short term, and therefore these agents have been labelled as having either bronchodilator or antibronchoconstrictor effects in COPD patients [132, 133].

#### 5.3.2. Additional Potential Therapeutic Applications of CysLTR1 Antagonists

CysLTs also contribute to the immunopathogenesis of numerous inflammatory disorders involving multiple organ systems. Together with modest cysLTR1-mediated suppressive effects on neutrophils, the cysLTR1-independent anti-inflammatory activities of cysLTR1 antagonists are of potential therapeutic value in controlling harmful neutrophilic inflammation in many disorders including acute respiratory distress syndrome (ARDS) and systemic sepsis [134, 135]. Eosinophils and mast cells are also involved in the immunopathogenesis of eosinophilic gastroenteritis and systemic mastocytosis, respectively [136, 137], as are activated T cells in inflammatory bowel disease and atherosclerosis [137, 138]. Disorders other than asthma and allergic rhinitis in which cysLTR1 antagonists have been used as experimental therapy are listed in Table 3 [133, 134, 136–175]. The wide range of organ systems and pathological processes for which cysLTR1 antagonists are potentially useful is likely a reflection of their diverse anti-inflammatory activities. However, further research is needed to fully elucidate the role of cysLTR1 antagonists in managing these conditions.

## 6. Conclusions

CysLTs produced predominantly by cells of the innate immune system modulate host defences via their sensitizing and stimulatory effects on immune and inflammatory cells, as well as on various types of structural cells. However, if produced inappropriately and/or excessively, cysLTs contribute to the immunopathogenesis of acute and chronic inflammatory conditions of both infective and noninfective origin. Elucidation of the involvement of cysLTs in the immunopathogenesis of allergic rhinitis and some subtypes of bronchial asthma led to the development of cysLTR1 antagonists, which are used primarily in the therapy and prophylaxis of these conditions. The discovery that these agents also possess secondary, cysLTR1-independent anti-inflammatory activities has evoked an awareness of the broader therapeutic utility of these agents, although specific clinical applications remain to be established.

## Figures and Tables

**Table 1:** Proinflammatory interactions of cysteinyl leukotrienes with cells of the innate immune system.

Cell type	Proinflammatory activities triggered by cysLTs
Mast cells [6–8]*	↑ Ca^2+^ influx ↑ Proliferation ↑ Production of MIP-1*β*/CCL4

Monocytes/macrophages [9–16]	↑ Migration ↑ Production of ROS ↑ Release of MMP-9 ↑ Production of MCP-1 and IL-8 ↑ Production of VEGF

Eosinophils [17–20]	↑ Migration ↑ Adhesion to vascular endothelium and airway epithelium ↑ Release of eosinophil-derived neurotoxin and cationic protein

Dendritic cells [14, 21, 22]	↑ Ca^2+^ influx ↑ Production of IL-10 ↑ Production of IL-8

Neutrophils [23–25]	↑ Ca^2+^ influx ↑ Production of nitric oxide and ROS (modest effect) ↑ Sensitization for increased production of ROS and release of MMP-8 following exposure to a second stimulus

*denotes references cited in the text.

**Table 2:** Cysteinyl leukotriene receptor-1-independent anti-inflammatory activities of montelukast, pranlukast, and zafirlukast.

Anti-inflammatory activity	Cell type	Antagonist
↓ Activity of 5-lipoxygenase [26, 30, 31]*	Neutrophils, monocytes/macrophages	Montelukast and zafirlukast

Inhibition of cyclic nucleotide phosphodiesterases [26, 32]	Neutrophils (also described in airway smooth muscle cells)	Montelukast

↓ Activity of NF*κ*B [29, 34–36]	Monocytes/macrophages (also described in T cells, epithelial cells and endothelial cells)	Montelukast and pranlukast

Inhibition of microsomal prostaglandin E synthase-1 [37]	Monocytes/macrophages	Montelukast, pranlukast, and zafirlukast

↓ Eosinophil adhesion and migration [38, 39]	Eosinophils	Montelukast

↑ Activity of AMP-activated protein kinase [43]	Relevance to cells of the innate immune system not yet established	Pranlukast

Antagonism of P2Y receptors [31]	Monocytes/macrophages	Montelukast and zafirlukast

*denotes relevant references cited in the text.

**Table 3:** Pathological conditions involving multiple organ systems which may be amenable to therapy with montelukast, pranlukast, or zafirlukast.

System	Leukotriene receptor antagonist		
	Montelukast	Pranlukast	Zafirlukast
Respiratory	(i) Bronchiolitis obliterans [139] (ii) Acute lung injury (ARDS) [134, 140] (iii) Eosinophilic-mediated lung inflammation [141] (iv) Cystic fibrosis [142]	(i) Pulmonary fibrosis [143, 144] (ii) Lung remodeling [145] (iii) Diffuse panbronchiolitis [146]	(i) COPD [133, 147] (ii) Bronchiectasis [148]

Gastrointestinal	(i) Eosinophilic gastroenteritis [136] (ii) Eosinophilic esophagitis [149] (iii) Irritable bowel syndrome [137] (iv) Portal hypertension [150]	(i) Gastric mucosal protection [151] (ii) Primary biliary cirrhosis [152] (iii) NSAID-induced intestinal damage [153]	Hepatorenal syndrome [154]

Neurological		(i) Alzheimer's disease [155, 156] (ii) Epilepsy [157] (iii) Acute cerebral ischemia [158, 159] (iv) Hypothermia-induced brain injury [160]	Autoimmune encephalomyelitis [161]

Dermatological	(i) Atopic dermatitis [162] (ii) Idiopathic urticaria [163]		Psoriasis [164, 165]

Cardiovascular	(i) Hypoxic myocardium [166] (ii) Atherosclerosis [138]		

Immunological	Systemic mastocytosis [167]	Endotoxin-induced shock [168]	

Other	(i) Graves' orbitopathy [169] (ii) Obstructive sleep apnea [170] (iii) Allergic conjunctivitis [137] (iv) Interstitial cystitis [137]	(i) Metastatic disease [171] (ii) Otitis media [172]	(i) Capsular contracture [173] (ii) Prostatitis [174] (iii) Candidiasis [175]

The relevant references are cited in parentheses.
